# Differential Cannabinoid Receptor Expression during Reactive Gliosis: a Possible Implication for a Nonpsychotropic Neuroprotection

**DOI:** 10.1100/tsw.2009.31

**Published:** 2009-03-31

**Authors:** Daniele De Filippis, Antonio Steardo, Alessandra D'Amico, Caterina Scuderi, Mariateresa Cipriano, Giuseppe Esposito, Teresa Iuvone

**Affiliations:** ^1^ Endocannabinoid Research Group, Department of Experimental Pharmacology, University of Naples Federico II, Italy; ^2^ Department of Pharmaceutical Sciences, University of Salerno, Italy; ^3^ Department of Human Physiology and Pharmacology `V. Erspamer', Sapienza University of Rome, Italy

**Keywords:** cannabinoid, reactive gliosis, neurodegenerative diseases

## Abstract

Activated microglia and astrocytes produce a large number of inflammatory and neurotoxic substances in various brain pathologies, above all during neurodegenerative disorders. In the search for new neuroprotective compounds, interest has turned to marijuana derivatives, since in several *in vitro*, *in vivo*, and clinical studies, they have shown a great ability to control neuroinflammation.

Despite the emerging evidence regarding pharmacological activities of cannabinoids, their effective introduction into clinical therapy still remains controversial and strongly limited by their unavoidable psychotropicity. Since the psychotropic effect of cannabinoids is generally linked to the activation of the CB1 receptor on neurons, the aim of our review is to clarify the function of the two cannabinoid receptors on glial cells and the differential role played by them, highlighting the emerging evidence of a CB_2_-mediated control of neuroinflammation that could liberate cannabinoids from the slavery of their central side effects.

## REACTIVE GLIOSIS

Initially known as "neuroinflammation", today it has become commonplace to hear neuroscientists speaking about "reactive gliosis" to describe the endogenous responses of the central nervous system (CNS) to injury. Reactive gliosis is specifically regarded as the accumulation of enlarged glial cells, intensely releasing inflammatory mediators, occurring immediately after CNS insult[1].

Glial cells, commonly called neuroglia or simply glia (Greek for "glue"), are non-neuronal cells that provide support and nutrition, maintain homeostasis, form myelin, and participate in signal transmission in the nervous system[2]. In the human brain, glia are roughly divided into astrocytes, oligodendrocytes, ependymal cells, microglia, and radial glia. It is estimated that glia outnumber neurons by about 10 to 1. It is inaccurate to consider glia merely as "glue" in the nervous system as the name implies, rather it is more of a "partner" to neurons. They are also crucial in the development of the nervous system and in processes such as synaptic plasticity and synaptogenesis[3]. Moreover, recent studies have evidenced the dark side of the glia. Chronic microglial activation, in fact, is an important component of neurodegenerative diseases[4], and this chronic neuroinflammatory component likely contributes to neuronal dysfunction, injury, loss, and, hence, to disease onset and progression[5]. The recognition of microglia as the brain's intrinsic immune system[6] and the understanding that chronic activation of this system leads to pathologic sequelae[7] has led to the modern concept of neuroinflammation[8]. This vision of microglia-driven neuroinflammatory responses, accounting for neuropathological consequences, has extended the older consideration of passive glial responses that are inherent in the concept of "reactive gliosis". In contrast, glial activation implies a more aggressive role in responding to activating stimuli, i.e., activated glial cells release factors that, in turn, self-perpetuate reactive gliosis affecting neighboring neuron viability. Activated microglia are thus considered as a common hallmark of many brain pathologies, including trauma and cerebral ischemia[9]. Much attention has been focused toward clarification of the detrimental role of several proinflammatory inducible proteins (iNOS, COX-2) and their products (NO, prostaglandins), together with interleukins (IL-1, IL-6) and chemokines (IL-8) over-released by activated glia, because these agents have been shown to prompt brain damage[10].

## TREATMENTS

Since the well-recognized role exerted by reactive gliosis is to sustain brain damage, scientists are constantly in search of new molecules therapeutically able to blunt this process, in an attempt to delay the progression of neurodegeneration.

Until now, only some benefits were obtained by the use of anti-inflammatory drugs, such as aspirin[11] or COX-2 inhibitors[12], or by the use of antioxidant compounds, such as melatonin[13] or vitamin E[14]. In the search for more efficient molecules, interest has turned to marijuana derivatives, since several *in vitro, in vivo,* and clinical studies have shown them to possess a great ability to manage neuroinflammation[15].

## CANNABINOIDS

Actually, the term "cannabinoids" (CBs) has been used to identify a wide range of natural terpenophenolic compounds isolated by *Cannabis sativa,* usually exhibiting pharmacological activity on specific receptor interactions. Two membrane receptors for CBs, both coupled to G protein and named $CB_1$ and $CB_2$, have been identified so far. While $CB_1$ receptors are mainly expressed in the CNS and the peripheral nervous system (PNS), $CB_2$ receptors are most abundant in cells of the immune system[16]. In the last 2 decades, specific endogenous ligands for CB receptors, producing similar but not identical effects to natural CBs, have been discovered and termed endocannabinoids[17]. Starting with the isolation of anandamide (AEA) and 2-arachidonoilglycerol (2-AG)[18], ethanolamide and ester of arachidonic acid, respectively, the endocannabinoid clan has been successively enlarged by identification of other molecules, including noladin ether[19] and oleamide[20].

Cannabis pharmacology is constantly expanding and therapeutic properties of CB agonists include analgesia, muscle relaxation, immunosuppression, anti-inflammatory and antiallergic effects, improvement of mood, stimulation of appetite, antiemesis, lowering of intraocular pressure, bronchodilation, neuroprotection, and antineoplastic effects (for review see [21]).

For the most part, CB attractiveness comes from their ability to maintain the homeostasis within both the CNS and PNS, through the neutralization of free radical species, prevention of cytotoxicity, hence to restore neuronal plasticity. Besides their direct neuroprotective actions, CBs blunt neuroinflammation, thus accounting for rebound neuroprotection. CBs display neuroprotective actions in both a CB receptor–dependent and –independent manner. Despite the emerging evidence regarding pharmacological activities of CBs, however, their effective introduction into clinical therapy is controversial and strongly limited by their unavoidable psychotropic effects. Since these actions are generally linked to the activation of $CB_1$ receptors on neurons, the aim of our review is to clarify the function of the two CB receptors on glial cells, emphasizing the emerging role of a $CB_2$-mediated control of neuroinflammation that could liberate CBs from the slavery of psycotropicity.

## FUNCTION OF $CB_1$ RECEPTORS

At the beginning of CB history, all their effects during neuroinflammation were commonly linked to the activation of $CB_1$ receptors, since at that moment, the presence of this receptor was only identified within the brain, while it was considered that glial cells were not endowed with $CB_2$ receptors[22].

It has been demonstrated that the anti-inflammatory effects of AEA depend on $CB_1$ receptor activation in LPS/IFN-γ–challenged astrocytes[22]. These findings have been confirmed by the evidence that selective $CB_1$ receptor agonism accounts for iNOS protein inhibition and NO release reduction in C6 rat glioma cells exposed to different neurotoxic stimuli[23,24].

In a more recent paper, selective $CB_1$ receptor activation was responsible for S100B protein down-regulation and consequent "rebound" neuroprotection, in a model of neurotoxicity due to MPTP exposure[25].

The importance of $CB_1$ receptors was strengthened by the observation that an *in vitro* model of β-amyloid (Aβ)–induced neuroinflammation agonism at this site is able to reduce NO, thus significantly contributing to the tau protein hyperphosphorylation rate decrease[26]. This evidence is in line with *in vivo* studies demonstrating that $CB_1$ receptor activation is able to attenuate Aβ-induced reactive gliosis, as proved by the decrease of GFAP and S100B, two pivotal glial markers. In addition, the CB-dependent reduction of S100B results in the prevention of glial proliferation, beneficially impacting the disease course[27].

Neuroinflammation profoundly alters the endocannabinoid system (ECS). In microglial cells, it has been demonstrated that AEA is able to induce, via the $CB_1$ receptor, the expression of mitogen-activated protein kinase phosphatase-1 (MKP-1) with consequent MAPK signal transduction attenuation[28]. Another study demonstrated that 2-AG, whose expression is increased after brain trauma, may have a neuroprotective role in reducing the accumulation of harmful mediators that may lead to secondary damage[29].

ECS alterations are very evident in Aβ-induced neuroinflammation. In fact, *in vitro* and *in vivo* evidence has shown an opposing control of CB receptor stimulation on Aβ-induced reactive gliosis; the $CB_1$ receptor and its preferential ligand AEA are significantly down-regulated while, on the contrary, $CB_2$ receptor expression and 2-AG release results are markedly increased[27].

The effects of CBs on glial cells were not only linked to the control of glial activation since, interestingly, recent data have evaluated their involvement on astroglial excitatory amino acid (EAA) transport[30]. The CB-induced EAA transport inhibition was partially blocked by the $CB_1$ receptor antagonist, proposing endocannabinoids as a novel class of inhibitors of the astroglial glutamate transport system.

## FUNCTION OF $CB_2$ RECEPTORS

At first, the influence of $CB_2$-mediated effects during neuroinflammation was disregarded due to the lack of their expression in primary astrocytes[31]. Other studies described the effect of the selective $CB_2$ antagonist SR144528, also in the absence of $CB_2$ receptors, probably due to the presence of a putative $CB_2$-like receptor[32]. The conclusive evidence showing the presence of $CB_2$ receptors in astrocytes still remains controversial, since a recent study characterized them in a constitutive manner in human astrocytes[33]. Moreover, recent observations demonstrate that a $CB_1/CB_2$ agonist, WIN-55212-2, prevents microglial cell activation during LPS-induced chronic neuroinflammation in young rats and worsens the LPS-induced impairment of performance in the water-maze test[34]. The effects of this agonist were not dependent on direct $CB_1$ receptor stimulation of microglia or astrocytes, suggesting an indirect effect of WIN-55212-2, or even better, a $CB_2$ involvement, on microglia activation and memory impairment[34].

The hypothesis that $CB_2$ receptors are also involved in the control of glial inflammation arises from the detection of both $CB_1$ and $CB_2$ receptors in stimulated microglia by RT-PCR[35]. In the same study, it was also found that only $CB_2$ receptors are involved in the antiviral activity of WIN-55212-2 on microglia[35].

It is now well recognized that $CB_2$ glial expression undergoes modulatory changes depending on cell activation state. $CB_2$ mRNA was not detected in total RNA obtained from whole adult or neonatal rat brains. However, it was detected in total RNA extracted from microglia maintained in culture for 1 or 24 h after isolation from mixed glial cultures[36]. Relatively low expression of $CB_2$ mRNA has been found in total RNA from mixed glia or from astrocytes. On the other hand, high $CB_2$ density was found in nonimmune-mediated pathological conditions, as well as during immune-mediated CNS pathology[36]. In mice with experimental autoimmune encephalomyelitis (EAE), $CB_2$ receptor mRNA was found to be increased 100-fold compared to healthy mice[36]. Moreover, microglial cells specifically present $CB_2$ receptors during EAE and their expression peaks up to tenfold in comparison to resting microglia[37]. A comparable pattern of differential $CB_2$ expression was also detected after Aβ insult, where a pronounced enhancement of the $CB_2$ receptor was evident both in C6 rat astroglioma cells and in rat hippocampal glia[27].

These results are in line with the observation that $CB_2$ receptors are increased in astrocyte-associated plaques from postmortem brains of Alzheimer's disease patients[38].

Although the localization of $CB_2$ receptors on glial cells is well known, their role during physiological and pathological conditions still remains to be clarified since, to date, conflicting evidence has emerged.

While microglial $CB_2$ activation appears protective against Aβ insult, contrarily, its expression by astrocytes leads to the aggravation of the reactive gliosis triggered by Aβ. In different experimental models of neurodegeneration, including Alzheimer's disease, Huntington's chorea, and amyotrophic lateral sclerosis, the activation of $CB_2$ receptors has been linked to a delayed progression of neuropathology. On the other hand, $CB_2$ detrimental effects were highlighted in Aβ-injected rats where the $CB_2$ agonist worsened the reactive gliosis course, while the antagonism at the same site attenuated the expression of S100B and GFAP significantly ameliorated neuroinflammation[27].

## CONCLUSION

The evidence that glial cells produce AEA, 2-AG, and other acetylethanolamines; express CB receptors; and also possess the molecular apparatus to inactivate these substances[39] suggested a functional endocannabinoid signaling system in these cells[40]. Likely, a relationship between the ECS and glial cells persuaded researchers to investigate the effects of its pharmacological manipulation on glial functions. We have attempted, in the present review, to summarize the main pharmacological and biochemical data showing the results reached until now concerning the role of CBs during glial inflammation. We have put particular emphasis on the different functioning of CB receptors. So far, the largest amount of data regards the activation of $CB_1$ receptors, since they were first discovered in the brain, and are also known as the CB central subtype. Even if the beneficial role of $CB_1$ activation during several proinflammatory insults is largely recognized, from bacterial LPS to neurotoxins (including MPTP or Aβ), in parallel, it was also established that during neuroinflammation, $CB_1$ tone is generally decreased as well as AEA, its preferential endocannabinoid ligand, losing in this way the biological control effected in neuroinflammation. Nevertheless, the use of direct $CB_1$ receptor agonists is strongly hampered by a variety of unwanted consequences, which include psychotropic effects, memory impairment, and mood alterations.

On the other hand, the exploit of $CB_2$ receptor subtypes remained unexplored for a long time, due to the inaccurate conviction of their absence in the brain, specifically on glia. It is now clear that $CB_2$ receptor expression in glial cells changes in relation to the cell activation state and, if it appears undetectable in resting cells, contrarily it drastically raises up after inflammatory stimuli. For this reason, the $CB_2$ receptor could be a perfect candidate for the control of glial inflammatory response, but unfortunately its activation has produced some controversial results during neuroinflammatory processes, alternating protective and detrimental effects. Although further investigations will be necessary to better elucidate the molecular mechanisms underlying the controversial effects of $CB_2$ activation on glial cells, the primary role played by this receptor still remains evident in the control of glial cell functions, which are compromised during inflammatory events and, thus, are crucial to ensure an optimal environment for neuronal survival and functioning.

In conclusion, pharmacological interactions at glial $CB_1$ and $CB_2$ receptors result in a marked regulation of reactive gliosis. To date, the more promising results have been reached by the use of compounds that selectively block $CB_2$ receptors, which may also have therapeutic value for their effects devoid of psychotropic consequences.

## ACKNOWLEDGMENT

This work was supported by FIRB - Piattaforme Reti 2006. We are grateful to the International Cannabinoid Research Society (ICRS) and to the National Institute on Drug Abuse (NIDA) for the travel award to Dr. De Filippis.
